# Electrochemical Investigation of Interfacial Properties of Ti_3_C_2_T_**x**_ MXene Modified by Aryldiazonium Betaine Derivatives

**DOI:** 10.3389/fchem.2020.00553

**Published:** 2020-07-24

**Authors:** Lenka Lorencova, Veronika Gajdosova, Stefania Hroncekova, Tomas Bertok, Monika Jerigova, Dusan Velic, Patrik Sobolciak, Igor Krupa, Peter Kasak, Jan Tkac

**Affiliations:** ^1^Institute of Chemistry, Slovak Academy of Sciences, Bratislava, Slovakia; ^2^Department of Physical Chemistry, Faculty of Natural Sciences, Comenius University, Bratislava, Slovakia; ^3^International Laser Centre, Bratislava, Slovakia; ^4^Center for Advanced Materials, Qatar University, Doha, Qatar

**Keywords:** carboxybetaine, sulfobetaine, Ti_3_C_2_T_**x**_ MXene, EIS, electrochemistry, zwitterions

## Abstract

For efficient and effective utilization of MXene such as biosensing or advanced applications, interfacial modification of MXene needs to be considered. To this end, we describe modification of Ti_3_C_2_T_x_ MXene by aryldiazonium-based grafting with derivatives bearing a sulfo- (SB) or carboxy- (CB) betaine pendant moiety. Since MXene contains free electrons, betaine derivatives could be grafted to MXene spontaneously. Kinetics of spontaneous grafting of SB and CB toward MXene was electrochemically examined in two different ways, and such experiments confirmed much quicker spontaneous SB grafting compared to spontaneous CB grafting. Moreover, a wide range of electrochemical methods investigating non-Faradaic and Faradaic redox behavior also in the presence of two redox probes together with contact-angle measurements and secondary ion mass spectrometry (SIMS) confirmed substantial differences in formation and interfacial presentation of betaine layers, when spontaneously grafted on MXene. Besides spontaneous grafting of CB and SB toward MXene, also electrochemical grafting by a redox trigger was performed. Results suggest that electrochemical grafting provides a denser layer of SB and CB on the MXene interface compared to spontaneous grafting of SB and CB. Moreover, an electrochemically grafted SB layer offers much lower interfacial resistance and an electrochemically active surface area compared to an electrochemically grafted CB layer. Thus, by adjusting the SB/CB ratio in the solution during electrochemical grafting, it is possible to effectively tune the redox behavior of an MXene-modified interface. Finally, electrochemically grafted CB and SB layers on MXene were evaluated against non-specific protein binding and compared to the anti-fouling behavior of an unmodified MXene interface.

## Introduction

Since their discovery in 2011 (Naguib et al., 2011), MXenes as 2D derivatives of MAX phases have been implemented in numerous applications due to their adsorption ability, hydrophilicity, large surface area, and high surface reactivity (Huang et al., 2020; Zhan et al., 2020; Zhang et al., 2020). Several breakthroughs in MXene synthesis and MXene-nanocomposite preparation resulted in various elemental compositions and surface functional tunabilities (Khazaei et al., 2019). During the last few years, MXenes have played an ever important role in terms of both crystalline and composition varieties for catalysis (Zhang et al., 2018; Benchakar et al., 2019; Li and Wu, 2019; Sun et al., 2019), environmental applications (Rasool et al., 2019), development of Li batteries (Ostadhossein et al., 2019; Wu Y.-Y. et al., 2019), and (bio)sensors (Kalambate et al., 2019; Lorencova et al., 2019; Mohammadniaei et al., 2019; Wu Q. et al., 2019; Gajdosova et al., 2020; Szuplewska et al., 2020).

In our recent review paper, we showed that although there are numerous enzyme-based electrochemical biosensors, development of affinity-based electrochemical biosensors is still in its infancy (Lorencova et al., 2019). We believe that in order to successfully develop sensitive and selective affinity-based electrochemical biosensors, particular attention needs to be paid to interfacial modification of MXene (Lorencova et al., 2019). Such interfacial patterning can be done using aryldiazonium salt-based grafting (Mahouche-Chergui et al., 2011; Wang et al., 2016; Cao et al., 2017) or using photoimmobilization for biopolymer grafting (Chocholova et al., 2018).

Zwitterionic modification of surface material containing derivatives with a highly ionic, charged balanced character is a benchmark for improvement of anti-biofouling properties. Betaines as derivatives with a zwitterionic nature are named according to their negatively charged moieties such as carboxybetaine, sulfobetaine, and phosphobetaine covalently linked to positively charged quaternary ammonium moieties within one molecule. Modification with such derivatives has been utilized widely by others and by us in order to improve performance and operational parameters of biosensors (Bertok et al., 2013, 2015), for protein stabilization (Kasák et al., 2016; Liu and Jiang, 2016), to develop blood-compatible materials (Chocholova et al., 2018) and for other advanced applications (Ilčíková et al., 2015; Zavahir et al., 2019).

In our recent paper, we showed that spontaneous immobilization of zwitterionic sulfo- (SB) and carboxybetaine- (CB) based molecules via aryldiazonium salt grafting is possible for interfaces containing free plasmons (a free electron cloud; Bertok et al., 2019b).

In this paper, we investigated if spontaneous grafting of aryldiazonium containing betaines (CB and SB) is possible. We proved that spontaneous grafting of CB and SB is achievable on MXene, and then we studied kinetics of spontaneous grafting of CB and SB on MXene. We examined how effective spontaneous grafting of SB and CB is on MXene compared to electrochemical grafting of CB and SB layers on MXene. Interfacial properties of spontaneous and electrochemical grafting of CB and SB were characterized electrochemically using various techniques, using contact-angle measurements and SIMS. Importantly, such a modification provides a dramatic reduction in non-specific protein adsorption compared to MXene. We believe that by grafting of betaine derivatives to MXene modified interfaces, betaines were covalently grafted.

## Experimental Section

### Reagents

Chemicals [i.e., acetonitrile, (CH_3_)_3_CONO, HBF_4_, KCl, K_3_(Fe(CN)_6_), K_4_(Fe(CN)_6_)·3H_2_O, Ru(NH_3_)_6_Cl_3_], bovine serum albumin (BSA), *N*-hydroxysuccinimide (NHS), *N*-(3-dimethylaminopropyl)-*N*′-ethylcarbodiimide hydrochloride (EDC), and phosphate buffer (PB) components (KH_2_PO_4_ and K_2_HPO_4_, pH 7.0) were of ≥99% purity or p.a. grade and were purchased from Sigma-Aldrich (Germany). MAX phase Ti_3_AlC_2_ was purchased from Carbon, Ltd., Kiev, Ukraine, with a declared size of particles of <100 μm and purity of 98 wt.%. Hydrochloric acid (HCl, 35–38 wt.%) was provided from Research-Lab Fine Chem Industries, Mumbai (India). Lithium fluoride (LiF, > 99.9 wt.%) was provided from Sigma-Aldrich (Germany). All solutions were made in ultrapure deionized water (DW, 18.2 MΩ·cm). In an effort to avoid oxidation of a prepared product, a dispersion of MXene was purged with nitrogen (99.99%, Air Liquid, Slovakia) during all processing steps.

### Synthesis of Ti_3_C_2_T_x_ MXene

Ti_3_C_2_T_x_ MXene (termed as MXene in the following text) was synthesized by the following few steps as described in our earlier study (Lorencova et al., 2018). See also Supplementary Material file for further details about synthesis of MXene and its characterization (Figures S1, S2).

### Synthesis of Betaines

**Scheme 1:**
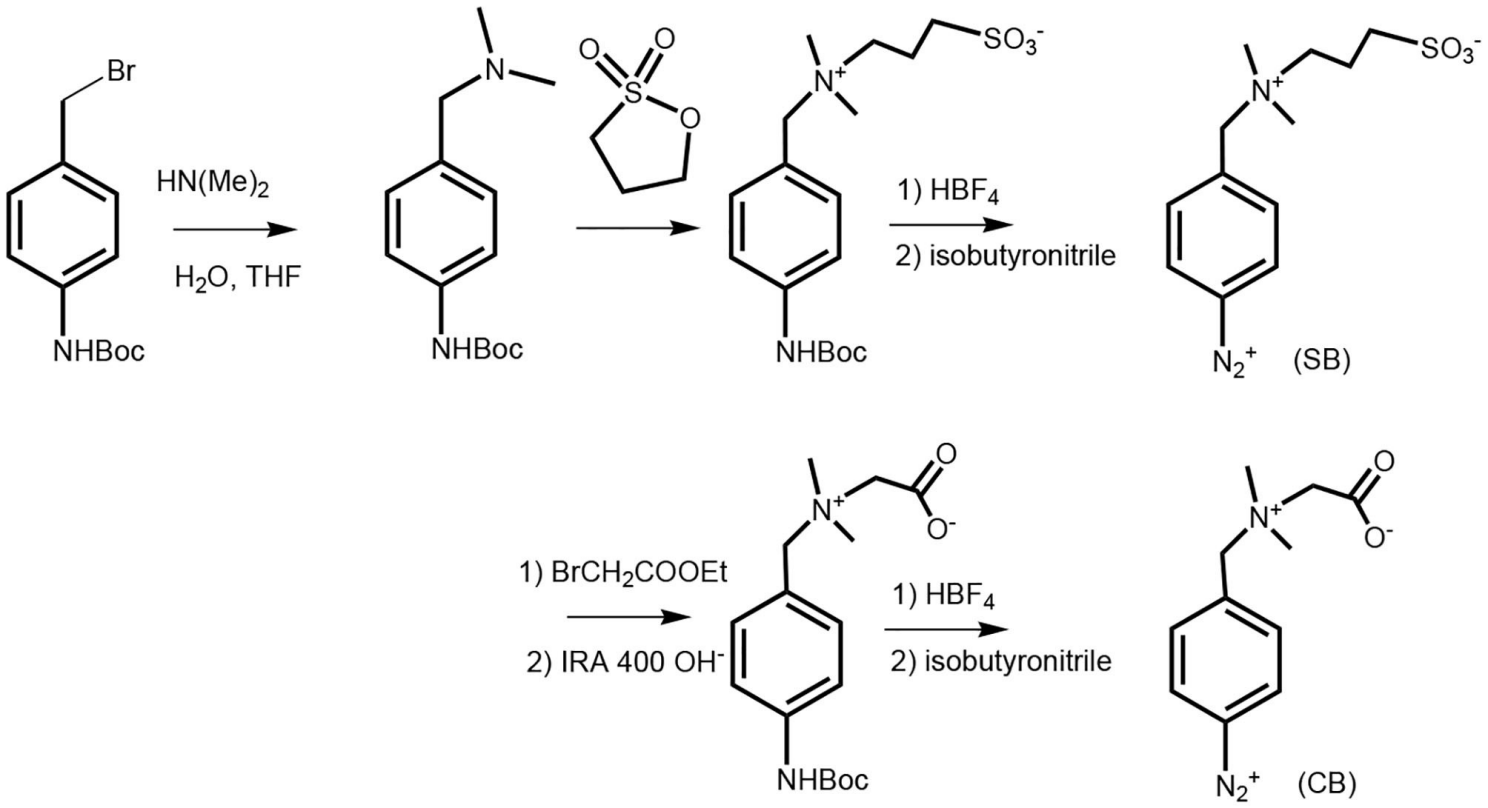
The schematic representation for synthesis of zwitterionic SB and CB derivatives; *tert*-butyl 4-(bromomethyl)phenylcarbamate was let to react with dimethylamine to form a dimethylamino derivative. For the synthesis of sulfobetaine, this derivative was let to react with 1,3-propanesultone forming a zwitterion derivative, which was (the same way as carboxybetaine) *in situ* deprotected under acidic conditions and form a diazonium salt. This derivative was applied for reaction with a working electrode surface in a spontaneous manner and using CV technique in a negative potential range to graft it electrochemically (a negative redox trigger). The carboxybetaine derivative synthesis is shown in the lower part of the scheme, where the dimethyl amino derivative was quarterized with ethyl bromoacetate and after hydrolysis of the ethyl ester group on ion exchanger IRA 400 led to a derivative that was *in situ* deprotected, formed diazonium salt, and was then applied the same way as an SB derivative.

The details about synthesis of betaines and its subsequent characterization with NMR and XPS techniques are summarized in our previously published papers (Bertok et al., 2019a,b). Synthesis of *tert*-butyl 4-[(dimethylamino)methyl]phenylcarbamate (Derible et al., 2014) and consequent syntheses of both derivatives carboxybetaine (CB) and sulfobetaine (SB) together with relevant schemes are briefly seen in Scheme 1.

### Contact-Angle Measurements

Contact-angle measurements were realized on a portable instrument System E (Advex Instruments, Czech Republic) to reveal values of the contact angle for MXene and MXene/CB (or SB)-modified interfaces. DW was the testing liquid with the droplet volume of 1 μL. The value of a contact angle was obtained as an average value of assays performed using 3 droplets.

### Electrochemical Experiments

Electrochemistry was run on a laboratory potentiostat/galvanostat Autolab PGSTAT302N (Ecochemie, Utrecht, Netherlands) employing a rotating disc glassy carbon electrode (RDGCE, *d* = 3 mm, Bioanalytical Systems, USA) as a working electrode, a counter Pt electrode, and an Ag/AgCl/3 M KCl reference electrode (Bioanalytical Systems, USA). All assays were run under Nova Software 1.10, and data acquired were evaluated using OriginPro 9.0.

Electrochemical impedance spectroscopy (EIS) is a sensitive and rapid method providing characteristics of an interfacial layer using a redox probe. EIS was performed in an electrolyte containing 5 mM K_3_[Fe(CN)_6_] and K_4_[Fe(CN)_6_]·3H_2_O in 0.1 M PB, pH 7.0. The EIS analysis was run at 50 different frequencies (in the range from 0.1 Hz to 100 kHz). The results were presented in a form of a Nyquist plot, with an equivalent circuit R[Q(RW)] applied for data fitting.

The redox behavior of the electrodes was examined using an outer-sphere redox probe Ru(NH_3_)_6_Cl_3_ with a final concentration of 5 mM in 1 M KCl. The potential was swept from +100 mV to −600 mV and back to +100 mV with varying scan rates in the range from 0.1 to 0.9 V·s^−1^.

### RDGCE Modification With MXenes and Subsequently With Zwitterionic Aryldiazonium Derivatives

RDGCE was polished mechanically with 1.0 μm alumina slurry and sonicated in DW. RDGCE was subsequently dried using a pure nitrogen stream. The solution of MXene (1.0 mg mL^−1^) was sonicated for 1 min under N_2_ atmosphere with the goal to prepare homogeneous non-oxidized dispersion. Finally, RDGCE was modified with 15 μL of MXene dispersion by adsorption until dried at room temperature in a dustless environment.

Zwitterionic aryldiazonium salt derivatives were prepared in few steps by reaction with tetrafluoroboric acid (HBF_4_) and *tert*-butyl nitrite [(CH_3_)_3_CONO] in acetonitrile. At the earliest, 59 mM protected diazonium salt solution in DW (1 equivalent) was mixed with 4 equivalents of HBF_4_ in acetonitrile at RT for 45 min in the dark. Consequently, 1.1 equivalent of (CH_3_)_3_CONO was added dropwise to the mixture (on ice) and the solution was mixed for 2 h at RT. The final solution was immediately diluted in 0.1 M PB pH 7.0 to obtain 1 mM solution of zwitterionic aryldiazonium salt derivative. Finally, diazonium salts were directly applied for modification of RDGCE/MXene in the two ways, i.e., spontaneously or electrochemically.

Spontaneous grafted layers of aryldiazonium derivatives were prepared spontaneously onto RDGCE/MXene by a pipetting volume of 50 μL CB or SB onto the surface of electrodes. This reaction ran for a period of 60 min (RDGCE/MXene/CB and RDGCE/MXene/SB).

The RDGCE/MXene electrodes were electrochemically modified with CB or SB derivatives layers by applying a cyclic voltammetry method in the range of potential from 0 V to −1.0 V at a sweep rate of 0.25 V s^−1^ for 24 cycles (RDGCE/MXene/CB_e and RDGCE/MXene/SB_e).

### TOF SIMS Analysis

Secondary ion mass spectrometry (SIMS) employing a time-of-flight (TOF) analyzer was applied as a surface-sensitive and qualitative analytical tool considering ppm–ppb as the level of sensitivity. The ION-TOF SIMS IV spectrometer (ION-TOF, Muenster, Germany) was applied for SIMS experiments with details provided in our previously published works (Lorencova et al., 2017; Gajdosova et al., 2020).

## Results and Discussion

### Non-faradaic Basic Electrochemical Studies in a Plain Buffer

**Figure 1:**
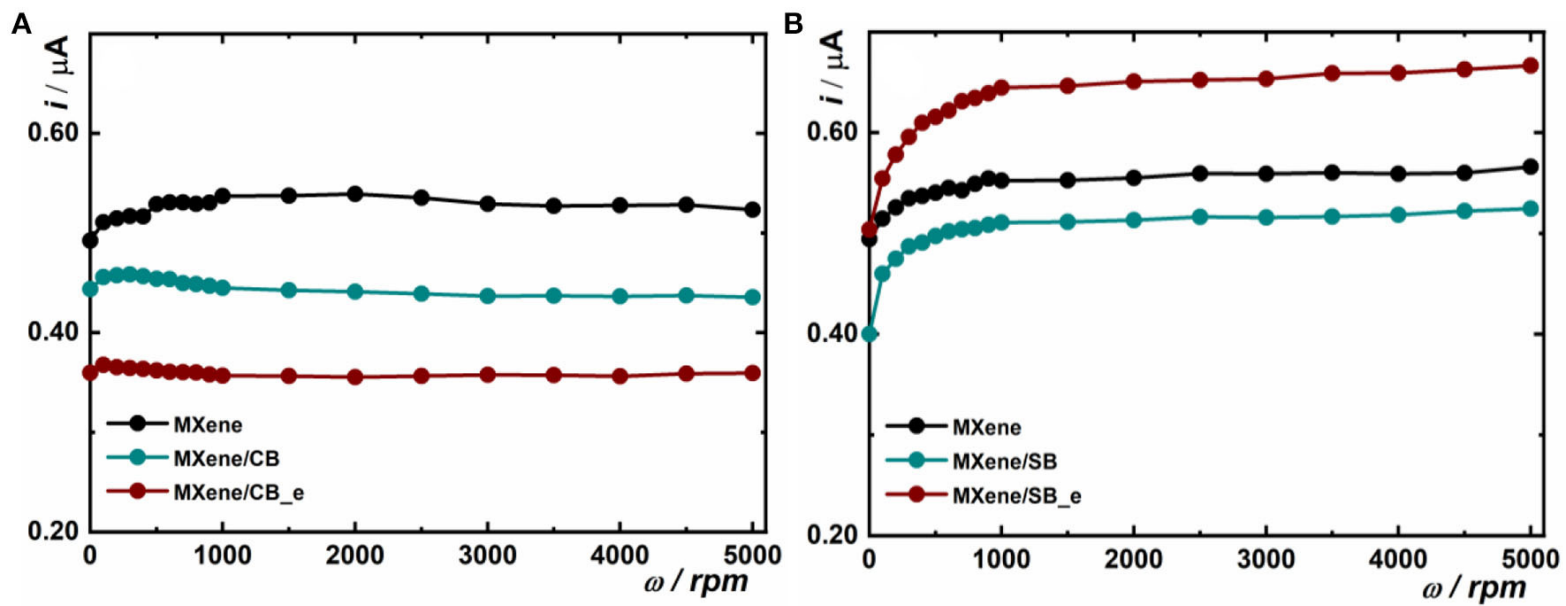
Plot of data extracted from the first electrochemical experiment. Anodic current read at a potential value of 0 V for every particular rotation speed applied to a modified RDGCE is shown. The experiment was run in 0.1 M PB pH 7.0 at a sweep rate of 100 mV s^−1^. The anodic current from CV read at 0 mV (see Figure S3) is plotted in the figure for CB **(A)** and SB **(B)** MXene modification.

The first electrochemical investigation was performed by running CVs in the plain 0.1 M PB pH 7.0 in a potential window from 0 V to −0.5 V with a sweep rate of 100 mV s^−1^ using RDGCE, which was rotated with a rotation speed ranging from 0 to 5,000 rpm (see Figure S3 as an example). The following electrodes were examined: RDGCE/MXene, RDGCE/MXene/CB, RDGCE/MXene/CB_e, RDGCE/MXene/SB, and RDGCE/MXene/SB_e. The results are summarized in Figure 1.

On the RDGCE modified by MXene and on the electrodes modified by MXene and CB, current decreased in order RDGCE/MXene > RDGCE/MXene/CB > RDGCE/MXene/CB_e (Figure 1A). Decrease in the capacitive current RDGCE/MXene vs. RDGCE/MXene/CB can be explained by presence of a CB layer. Further decrease in capacitive current on RDGCE/MXene/CB_e suggests further increase in CB density.

For SB-modified MXene, the highest capacitive current even higher than on the MXene-modified interface was observed on RDGCE/MXene/SB_e (Figure 1B). This suggests that RDGCE/MXene/SB_e exhibits a higher or comparable electrochemically active surface area, when compared to the MXene interface (Figure 1B).

It can be concluded that CB-modified MXene compared to an MXene interface modified by SB offered lower electrochemically active surface area and that interfacial behavior for CB and SB modified MXene is different.

### Contact-Angle Measurements

The contact-angle value dropped from 37° (RDGCE/MXene) to 20° (RDGCE/MXene/CB) and further to 17° (RDGCE/MXene/CB_e) (Table 1). This can also mean that electrochemically triggered CB grafting can increase the density of CB (RDGCE/MXene/CB_e) compared to spontaneous grafting of CB on MXene (RDGCE/MXene/CB). Alternatively, this might also mean that electrochemical CB grafting compared to spontaneous formation of CB on MXene resulted in the formation of a layer differing in the structure of the grafted molecule, grafting density, density of pinholes, and orientation of the grafted molecules.

**Table 1:** Contact-angle values on various interfaces.

**Electrode**	**Contact** **angle/**°****	**Electrode**	**Contact angle/**°****
RDGCE	102 ± 2	RDGCE	102 ± 2
RDGCE/MXene	37 ± 2	RDGCE/MXene	37 ± 2
RDGCE/MXene/CB	20 ± 2	RDGCE/MXene/SB	11 ± 1
RDGCE/MXene/CB_e	17 ± 3	RDGCE/MXene/SB_e	11 ± 1

The contact-angle value dropped from 37° (RDGCE/MXene) to 11° (both RDGCE/MXene/SB and RDGCE/MXene/SB_e). This suggests that upon electrochemically triggered SB grafting on MXene, the density of SB did not change that much compared to spontaneous SB grafting on MXene.

At the same time, we can conclude that SB-grafted MXene is more hydrophilic compared to CB-grafted MXene and this feature can explain why SB-modified MXene exhibits a higher electrochemically active surface area compared to CB-grafted MXene interfaces (Figure 1).

### Non-faradaic Electrochemistry to Study Kinetics of Spontaneous CB/SB Grafting on MXene

**Figure 2:**
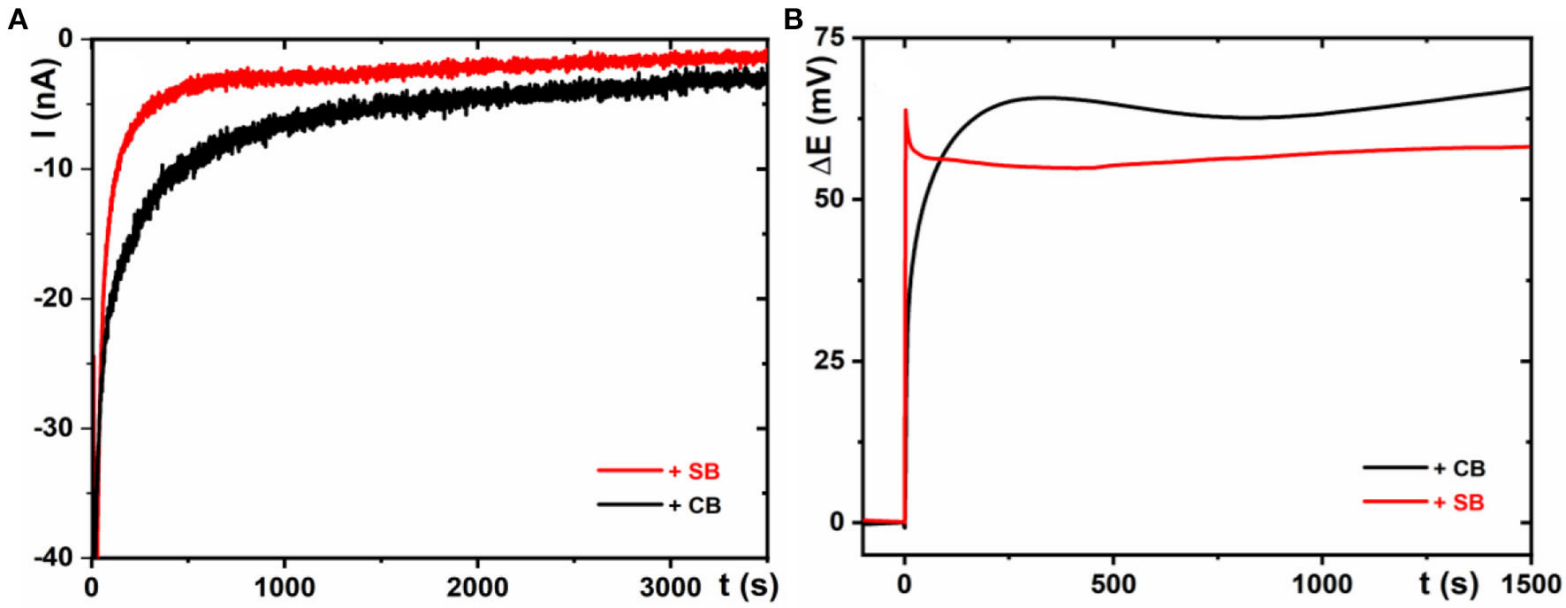
Chronoamperometric investigation of the spontaneous grafting of SB and CB on MXene **(A)** and an open-circuit measurement during spontaneous grafting of SB and CB on MXene **(B)**. Both zwitterions were added into the electrolyte to have a final CB/SB concentration of 1 mM. For other experimental conditions, see Figure 1.

Two different electrochemical experiments were performed to follow kinetics of spontaneous grafting of zwitterions onto MXene-modified electrodes. The first one was a chronoamperometric experiment examined by measurement of a change in current during addition of CB or SB into the electrochemical cell (Figure 2A). The second one was measurement of a change in the open-circuit potential during addition of CB or SB into the electrochemical cell (Figure 2B). Both experiments confirmed much quicker spontaneous grafting of SB over CB. The finding that spontaneous grafting of SB on MXene is much quicker than spontaneous grafting of CB on MXene might seem to be contradictive at first sight. The apparent contradiction might come from the following facts: MXene is negatively charged with a zeta potential of −30 mV at pH 7.0 (Ying et al., 2015; Shah et al., 2017; Chen et al., 2019) and SB has a permanent charge. Thus, it would be expected that SB will be repelled from a negatively charged MXene. The following facts can explain this apparent discrepancy: since a sulfo group is much bulkier compared to a carboxy group, a negative volumetric charge density is slightly higher for a carboxy group (~5.3 electrons *per* nm^3^) compared to a sulfo group (~4.5 electrons *per* nm^3^), while a positive volumetric charge density for quaternary amine is ~3.0 electrons *per* nm^3^ (Shao and Jiang, 2015). SB molecules can interact with each other more strongly *via* zwitterionic associations (i.e., pairing of the anionic and cationic groups) compared to CB molecules, and thus CB molecules have stronger interactions with external positively charged species compared to SB (Shao et al., 2014); a number of methylene groups separating cationic and anionic groups within these zwitterions do no play any role in this distinctive zwitterionic associations (Shao et al., 2014).

Thus, CB molecules are more negatively charged compared to SB molecules, and as a result, SB molecules can be spontaneously grafted to a negatively charged MXene at a higher speed and can reach higher interfacial density (see below).

### Faradaic Electrochemistry in a Plain Buffer

**Figure 3:**
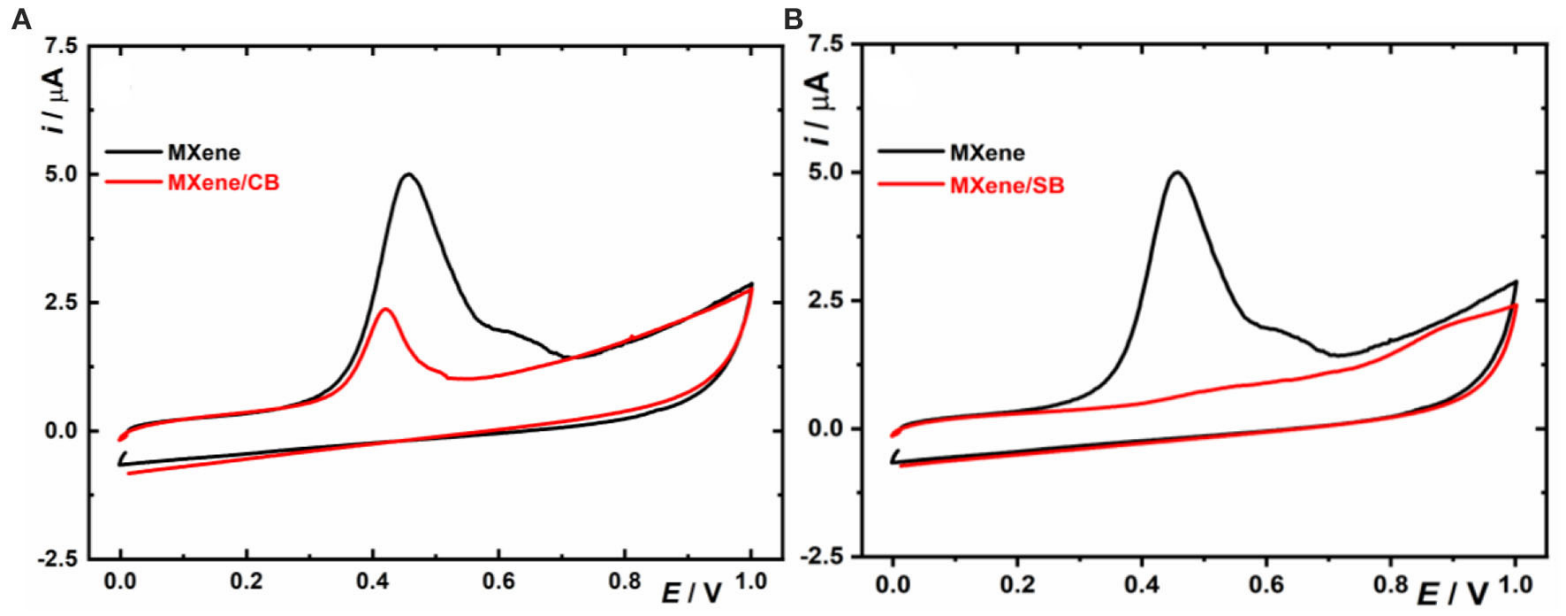
First scan of CVs shown for MXene and MXene modified with CB **(A)** and with SB **(B)**. For other experimental conditions, see Figure 1.

A typical anodic oxidation peak on MXene occurs, when exposing such interfaces to an applied voltage exceeding +0.4 V. It is quite interesting that upon spontaneous modification of MXene by CB, such oxidation peak is significantly lower compared to MXene (1.4 μC for MXene/CB and 6.0 μC for MXene) (Figure 3A). The difference between CV of MXene and MXene/SB is even more pronounced with only a capacitive peak observed on MXene/SB (Figure 3B). Spontaneous grafting of CB or SB onto the MXene-modified electrodes is possible only via free electrons present within MXene. Thus, we can suggest that an anodic peak might represent a density of free electrons in MXenes. At the same time, we can conclude that spontaneous formation of a denser MXene/SB layer compared to an MXene/CB layer was associated with a higher number of free electrons consumed for spontaneous formation of MXene/SB compared to MXene/CB.

This conclusion was made by taking into account that spontaneous SB grafting on MXene is more efficient (in terms of kinetics and degree of functionalization, see sections Non-Faradaic Electrochemistry to Study Kinetics of Spontaneous CB/SB Grafting on MXene and SIMS Experiments) compared to spontaneous CB grafting on MXene, and this is why a large number of free electrons are consumed by spontaneous SB grafting compared to spontaneous CB grafting.

### Faradaic Electrochemical Studies Using Redox Probes

#### Electrochemical Impedance Spectroscopy (EIS) Using a Ferri/Ferro Redox Couple

**Figure 4:**
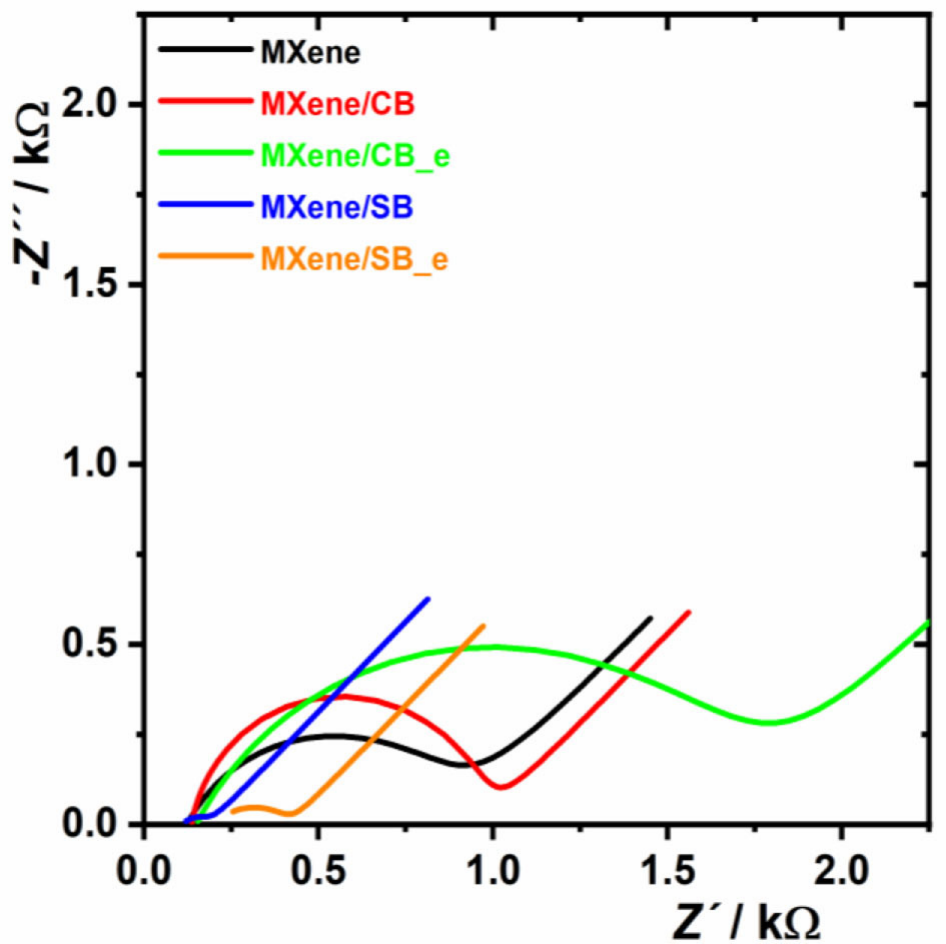
Electrochemical impedance spectroscopy investigated on MXene and zwitterion-modified MXene interfaces in 5 mM ferricyanide/ferrocyanide solution in 0.1 M PB pH 7.0. The EIS analysis was run at 50 different frequencies (in the range from 0.1 Hz to 100 kHz). The results were presented in a form of a Nyquist plot, with an equivalent circuit R[Q(RW)] applied for data fitting.

EIS using a ferri/ferro redox couple revealed that CB-modified MXene has higher charge transfer resistance R_ct_ (i.e., 838 Ω for MXene/CB and 1,610 Ω for MXene/CB_e) compared to unmodified MXene (786 Ω). Contrarily, SB-modified MXene has significantly lower R_ct_ (i.e., 88 Ω for MXene/SB and 228 Ω for MXene/SB_e) compared to unmodified MXene (786 Ω) and MXene modified by CB (Figure 4). This behavior is consistent with the discussion above regarding differences in the zwitterionic associations between SB and CB molecules with effective pairing of the anionic and cationic groups for SB and thus with low resistance toward a negatively charged redox probe. At the same time, it is worth mentioning that zwitterion-modified MXene prepared by a redox-triggered grafting showed higher R_ct_ compared to a spontaneous grafting for CB (1,610 vs. 838 Ω) and for SB (228 vs. 88 Ω). This is consistent with the higher density of grafted zwitterions induced by a redox trigger compared to spontaneously grafted zwitterions.

#### Electrochemistry With a Ru${{(\text{NH}}_{3})}_{6}^{3+}$ Redox Probe

When a Ru${{(\text{NH}}_{3})}_{6}^{3+}$ redox probe was applied for investigation of the interfacial behavior of unmodified and modified MXenes, a quite complex behavior was observed (Tables S1–S5).

The first interesting information obtained from such experiments is a formal redox potential of the redox probe found as follows: (−0.182 ± 0.003) V for MXene; (−0.137 ± 0.009) V for MXene/CB; (−0.172 ± 0.001) V for MXene/CB_e; (−0.123 ± 0.001) V for MXene/SB; and (−0.122 ± 0.001) V for MXene/SB_e. Thus, we can observe two different redox processes, i.e., at a potential of ~ −176 mV and at a higher redox potential of ~ −122 mV. A redox process occurring at a lower potential can be assigned to a surface-confined redox probe, while the one at a higher potential is associated with a diffusional electrochemistry of Ru${{(\text{NH}}_{3})}_{6}^{3+}$ (Campos and Ferapontova, 2014). Thus, the redox process of Ru${{(\text{NH}}_{3})}_{6}^{3+}$ on MXene and on MXene/CB can be assigned to a surface-confined redox probe. This can be ascribed to the fact that unmodified MXene is negatively charged with a zeta potential of −30 mV at pH 7.0 (Ying et al., 2015; Shah et al., 2017; Chen et al., 2019) and that MXene/CB_e should be strongly negatively charged, as well (see discussion in section Contact Angle Measurements).

A formal potential on MXene/SB and MXene/SB_e can be assigned to a diffusional electrochemistry of Ru${{(\text{NH}}_{3})}_{6}^{3+}$. Pairing of the anionic and cationic groups within the SB layer between neighboring SB molecules shielded a negative charge of sulfo group and thus Ru${{(\text{NH}}_{3})}_{6}^{3+}$ was not electrochemically confined on the interface.

The redox behavior of MXene/CB is in between these two redox processes based on the value of a formal potential of −137 mV, indicating simultaneous occurrence of homogeneous and surface confined electrochemistry of Ru${{(\text{NH}}_{3})}_{6}^{3+}$. At the same time, the value of a redox potential of −137 mV observed on MXene/CB is closer to a value of −122 mV than to −176 mV, indicating that the redox behavior of Ru${{(\text{NH}}_{3})}_{6}^{3+}$ on MXene/CB is dominated by a homogeneous electrochemistry of the redox probe rather than by a surface-confined redox process.

**Figure 5:**
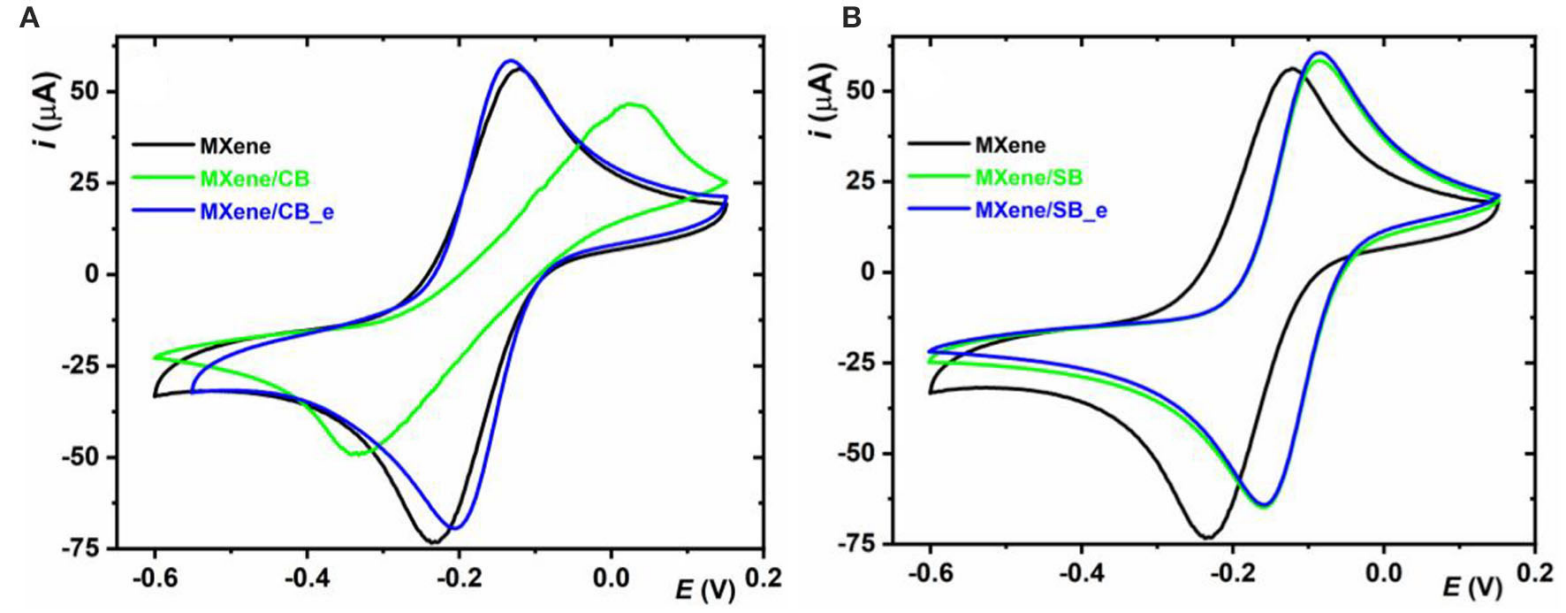
**(A)** and **(B)** Electrochemical behavior of of Ru${{(\text{NH}}_{3})}_{6}^{3+}$ investigated on MXene and zwitterion-modified MXene interfaces. Redox behavior of the electrodes was examined using an outer-sphere redox probe Ru(NH_3_)_6_Cl_3_ with a final concentration of 5 mM in 1 M KCl. The potential was swept from +150 mV to −600 mV and back to +150 mV with a scan rate of 0.1 V·s^−1^.

The other striking difference in the redox behavior of a Ru${{(\text{NH}}_{3})}_{6}^{3+}$ redox probe can be seen in CVs, which were run at a sweep rate of 100 mV s^−1^ (Figure 5). Electrochemistry of Ru${{(\text{NH}}_{3})}_{6}^{3+}$ on MXene/CB was irreversible with Δ*E* = 369 mV, while a much lower value of 115 mV was observed on MXene and 73 mV on MXene/CB_e, MXene/SB, and MXene/SB_e (Tables S1–S5). Irreversibility of Ru${{(\text{NH}}_{3})}_{6}^{3+}$ on MXene/CB is even more evident at a high sweep rate of 900 mV s^−1^ with Δ*E* = 581 mV (Table S2, Figure S4); Thus, by comparing *E*^0^ and Δ*E* values, we can confirm a quite complex redox behavior of a Ru${{(\text{NH}}_{3})}_{6}^{3+}$ redox probe on MXene/CB compared to other MXene-modified interfaces.

### SIMS Experiments

The redox behavior of MXene/CB in the presence of Ru${{(\text{NH}}_{3})}_{6}^{3+}$ can be explained by the fact that CB deposited spontaneously on MXene does not cover the MXene interface completely. The SIMS experiment indicated that CB deposited spontaneously on MXene covered only 36% of MXene, when compared to MXene/CB_e for the C_3_H_8_N^+^ ion fragment (Table 2). Analysis of two other ion fragments by SIMS indicated even lower surface coverage for CB deposited on MXene spontaneously, when compared to a redox-triggered grafting of CB (i.e., 34% for C_3_H_10_N_2_${\text{O}}_{2}^{+}$ and 25% for C_9_H_10_${\text{O}}_{2}^{+}$). This means that CB, which is in PB solution strongly associated with K^+^ (see discussion in section Contact Angle Measurements), could interact with MXene in a way that CB molecules deposited spontaneously are laying down on MXene. Thus, on one side –COOH associated with K^+^ is interacting with a negatively charged MXene via K^+^ and thus a positive charge from quaternary amine is exposed to the solution, repelling a positively charged redox probe from reaching the MXene surface. Of course, it is possible that on the MXene/CB interface there will be patches of CB molecules spontaneously grafted at quite a high density with CB molecules standing in an upright position with –COOH groups exposed to the solution phase. This is why on MXene/CB a mixed diffusional and surface-confined redox behavior of a Ru${{(\text{NH}}_{3})}_{6}^{3+}$ redox probe was observed.

**Table 2:** Results from SIMS investigation in a positive mode for selected three fragments/ions.

	**Intensities normalized to total intensity/cts/positive polarity** **×10**^****−3****^						
**Fragment**	**MXene A**	**MXene/CB B**	**MXene/CB_e C**	**B/C**	**MXene/SB D**	**MXene/SB_e E**	**D/E**
C_3_H_8_N^+^	0.87	24.1	67.1	0.36	18.6	31.2	0.60
C_3_H_10_N_2_${\text{O}}_{2}^{+}$	0.18	11.5	33.4	0.34	12.8	27.8	0.46
C_9_H_10_${\text{O}}_{2}^{+}$	0.28	13.5	54.6	0.25	12.7	35.9	0.35

*Data were extracted from Figures S10–S14 showing SIMS spectra*.

When CB was grafted on MXene by a redox trigger, CB molecules formed a dense layer of standing CB molecules with –COOH groups exposed to the solution phase. This is why such interface exhibited a surface-confined redox behavior and reversible electrochemistry for a Ru${{(\text{NH}}_{3})}_{6}^{3+}$ redox probe.

The situation with SB grafted on MXene is different compared to grafting of CB to MXene. SB molecules grafted to MXene spontaneously covered 35–60% of the surface of MXene, when compared to MXene/SB_e (Table 2). Thus, a quite high density of SB grafted to MXene spontaneously does not allow SB molecules to lay down on the interface with a preferential standing position of SB molecules on MXene. Besides, pairing of anionic and cationic groups of the neighboring SB molecules resulted in the interface only mildly negatively charged. This is why on MXene/SB and MXene/SB_e interfaces a reversible and diffusional redox behavior of a Ru${{(\text{NH}}_{3})}_{6}^{3+}$ redox probe was observed.

If we take into account that a surface coverage of spontaneously grafted SB on MXene compared to MXene/SB_e is only 35% (for C_9_H_10_${\text{O}}_{2}^{+}$ ion), it is possible that SB molecules can partly lay down on MXene not being in an upright standing position. Even in that situation, the distance between neighboring SB molecules and MXene interface should not be large enough to not allow pairing of anionic and cationic groups of the neighboring SB molecules laying on the interface. As a result, such SB-modified MXene interfaces, i.e., MXene/SB and MXene/SB_e, exhibited a diffusional and reversible redox behavior for a Ru${{(\text{NH}}_{3})}_{6}^{3+}$ redox probe.

An alternative explanation for the differences in the grafting between CB and SB might be the different ability to form multilayers during grafting of CB or SB layers, since grafting aryldiazonium-containing molecules to interfaces in most cases results in formation of multilayers (Berisha et al., 2015).

The fact that CB grafted on MXene spontaneously formed a layer, which was not that homogeneous compared to MXene/CB_e, can be confirmed by a SIMS 2D image (Figure S5) obtained from SIMS analysis.

A more detailed electrochemical analysis of MXene and MXene-modified interfaces in the presence of a Ru${{(\text{NH}}_{3})}_{6}^{3+}$ redox probe is shown in Figures S6–S9. Such an in-depth electrochemical analysis confirmed the fact that a redox behavior of a Ru${{(\text{NH}}_{3})}_{6}^{3+}$ redox probe on MXene/CB_e is similar to the redox behavior seen on MXene/SB and MXene/SB_e. The redox behavior on MXene/CB is distinct from the redox behavior of a Ru${{(\text{NH}}_{3})}_{6}^{3+}$ redox probe seen on MXene/CB_e, MXene/SB, and MXene/SB_e (Figures S6–S9).

### Non-specific Protein Binding to Unmodified and Modified MXene Interfaces

**Figure 6:**
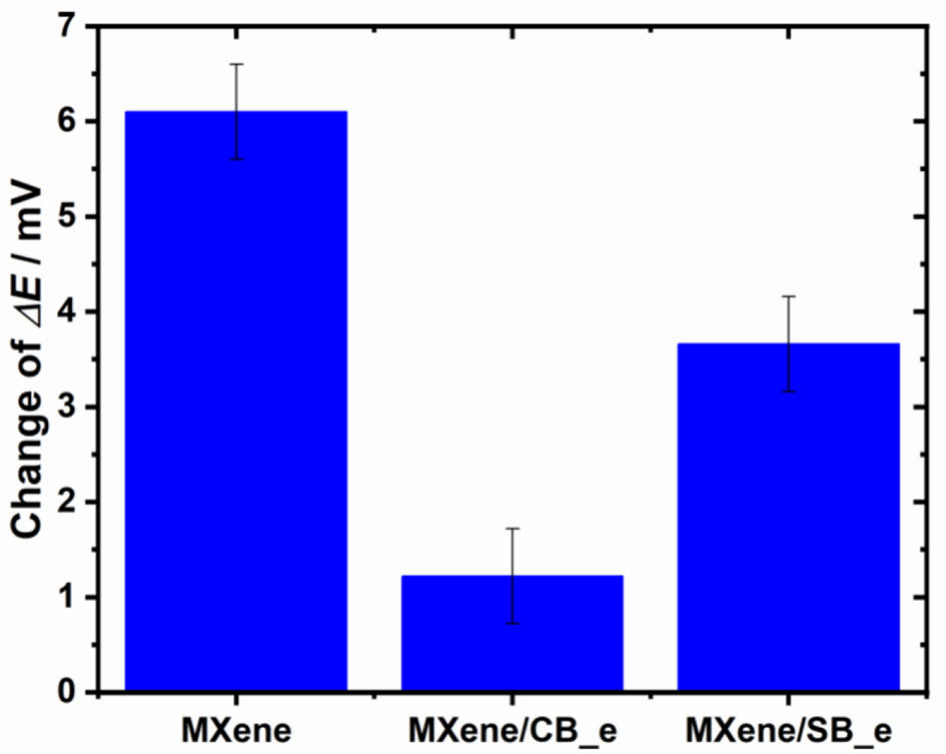
Investigation of a non-specific interaction of 100 nM BSA protein as a non-specific binding probe on various layers deposited on MXene using Ru${{(\text{NH}}_{3})}_{6}^{3+}$ as a redox probe. Non-specific binding was investigated as a change in Δ*E* of Ru${{(\text{NH}}_{3})}_{6}^{3+}$ after a 10-min incubation with the protein on all three MXene-modified interfaces.

In order to construct in the future any biosensor device, it is good to prepare a hybrid zwitterion interface consisting of CB and SB moieties. Dilution of CB by SB will allow to finely tune the density of –COOH groups applied for immobilization of biorecognition elements and thus allow to control interfacial density of biorecognition elements. From the results shown above, dilution of CB by SB will allow to tune also interfacial redox behavior such as redox behavior of the redox probe, interfacial resistance, and electrochemically active surface area. Thus, the biosensor construction could be optimized in a way to achieve high sensitivity of detection, while resisting non-specific protein binding from complex samples such human serum. The ability to resist non-specific protein binding on SB_e and CB_e modified MXene is shown in Figure 6, which shows that non-specific binding of 100 nM bovine serum albumin (BSA, a non-specific binding probe) expressed as a change in Δ*E* of a redox probe Ru${{(\text{NH}}_{3})}_{6}^{3+}$ on MXene was 6.1 ± 0.5 mV, while on GCE/MXene/CB_e a value of 1.2 ± 0.5 mV and on GCE/MXene/SB_e a value of 3.6 ± 0.5 mV were observed. Thus, a zwitterion grafter MXene surface exhibited significantly reduced non-specific protein binding, a feature very important for the development of affinity MXene-based biosensor devices.

## Conclusions

The results shown here indicate that various electrochemical techniques could be of a high added value for better understanding of the process of spontaneous grafting of CB and SB on MXene. In combination with other techniques such as contact-angle measurements and SIMS, we proposed a possible way how these layers were spontaneously developed on MXene. It is good point to the fact that rather inexpensive electrochemical infrastructure can render valuable data, which were further confirmed by other techniques such as contact-angle measurements and SIMS. The study is a solid foundation for further development of betaine-modified MXenes into functional enzyme and affinity-based electrochemical biosensors.

## Data Availability Statement

All datasets generated for this study are included in the article/Supplementary Material.

## Author Contributions

LL, TB, PK, and JT contributed conception and design of the study. LL, VG, SH, and TB conducted electrochemical experiments. MJ and DV did SIMS experiments. PS, IK, and PK prepared MXene and betaine derivatives. LL, PK, and JT wrote the manuscript. All authors contributed to manuscript revision, read, and approved the submitted version.

## Conflict of Interest

The authors declare that the research was conducted in the absence of any commercial or financial relationships that could be construed as a potential conflict of interest.
