# Supplementary material for: Electrochemical Investigation of Interfacial Properties of Ti3C2Tx MXene Modified by Aryldiazonium Betaine Derivatives
Source: Front Chem. 2020 Jul 24;8:553. doi: 10.3389/fchem.2020.00553 (PMC7393994; doi:10.3389/fchem.2020.00553)
Supplement: Supplementary file 1 [file Data_Sheet_1.PDF]

Supplementary Material

S1. Experimental section

$Ti_3C_2T_x$ was prepared by etching of Al from Ti_3AlC_2 (Fig. A) via in-situ produced HF through a reaction of LiF and HCl. MXene was prepared by etching of Al layer from 100 mg Ti_3AlC_2 in 50 mg mL^{-1} LiF solution (in 1.8 mL, concentrated HCl and water in 1:1 ratio) and magnetically stirred at 40 °C for 24 h. The produced multi-layered MXene was collected by centrifugation and extensively washed with DW. Delamination of MXene was performed by a probe sonication of multilayered MXene in degassed water, under inert gas, for 1 h. In the last step, the resulting solution was centrifuged at 3,500 rpm for 40 min and the supernatant was collected and freeze dried. $Ti_3C_2T_x$ MXene (MXene in the following text) was delaminated by an extensive sonication and single or few layered MXene was produced as seen from Fig. B. Delaminated MXene flakes were prepared with an average lateral dimension of $Ti_3C_2T_x$ of (59 ± 17) nm as seen from size distribution diagram (Fig. C).

An advantage of using LiF/HCl over HF was that both etching and intercalation were achieved in the exfoliation process, which significantly simplified the delamination of $Ti_3C_2T_x$ [1].

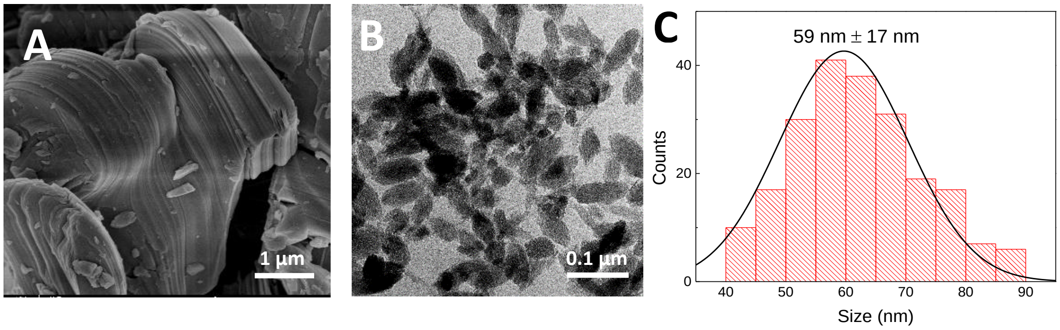

Figure S1: A) SEM of MAX phase, **B)** TEM of MXene and **C)** size distribution diagram of MXene.

XRD analysis was performed in order to confirm the removal of Al from Ti_3AlC_2 (Fig. S2). The characteristic (002) peak of Ti_3AlC_2 at 9.5° broadens and shifts to a lower value, what is caused by removing of Al and subsequent structural expansion due to substitution of Al with –F and –OH/O terminating groups, resulting in a larger d-spacing. In addition, the non-basal plane peaks of Ti_3AlC_2, most notably the (104) peak significantly lost on intensity for $Ti_3C_2T_x$. The (001) peaks, such as the (002), (004), and also (110) peak, broadened, decreased in intensity and shifted due to removing Al from the Ti_3AlC_2 [2].

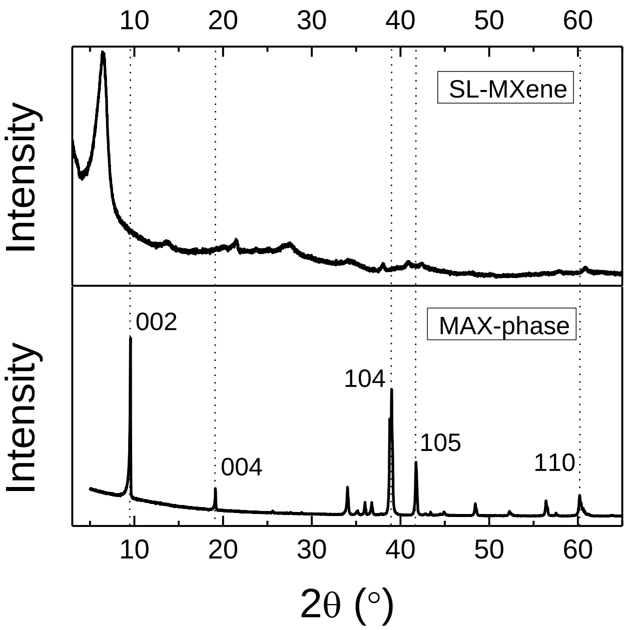

Figure S2: XRD of MXene (upper) and MAX phase (bottom).

S2. Results

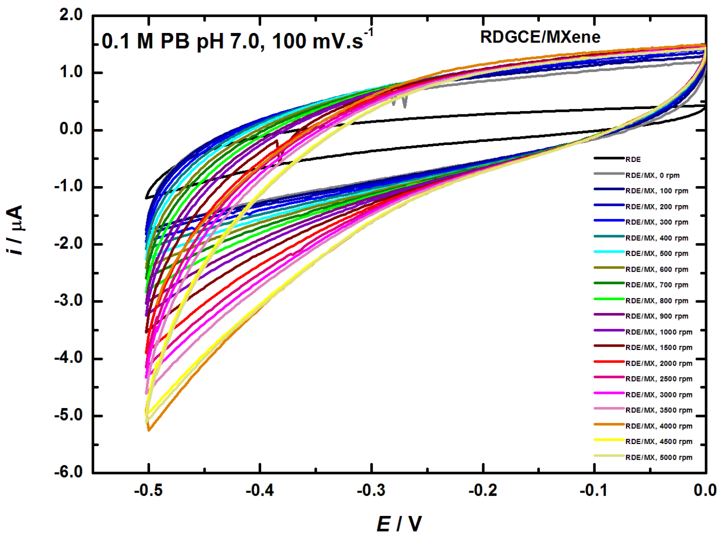

Figure S3: CVs run on MXene modified RDGCE by revolving at rotation speed ranging from 0 to 5,000 rpm. The experiment was run in 0.1 M PB pH 7.0 at a sweep rate of 100 mV s^{-1}.

Table S1: Electrochemical characteristics of a redox probe $Ru(NH_3)_6Cl_3$ on RDGCE/MXene

**MXene**							
**Scan rate / V s^{-1}**	**E_{pc} / V**	**E_{pa} / V**	**E^0 / V**	**ΔE / V**	**i_{pc} / µA**	**i_{pa} / µA**	**i_{pc} / i_{pa}**
**0.1**	-0.237	-0.122	-0.180	0.115	-73	56	1.31
**0.2**	-0.247	-0.112	-0.180	0.134	-106	85	1.24
**0.3**	-0.254	-0.105	-0.180	0.149	-131	107	1.22
**0.4**	-0.259	-0.103	-0.181	0.156	-153	127	1.21
**0.5**	-0.266	-0.090	-0.178	0.176	-174	143	1.21
**0.6**	-0.274	-0.088	-0.181	0.186	-192.	159	1.21
**0.7**	-0.283	-0.086	-0.184	0.198	-210	174	1.21
**0.8**	-0.291	-0.081	-0.186	0.210	-225	188	1.20
**0.9**	-0.296	-0.078	-0.187	0.217	-243	201	1.21

Table S2: Electrochemical characteristics of a redox probe $Ru(NH_3)_6Cl_3$ on RDGCE/MXene/CB

**MXene/CB**							
**Scan rate / V s^{-1}**	**E_{pc} / V**	**E_{pa} / V**	**E^0 / V**	**ΔE / V**	**i_{pc} / µA**	**i_{pa} / µA**	**i_{pc} / i_{pa}**
**0.1**	-0.342	0.027	-0.158	0.369	-49	47.0	1.06
**0.2**	-0.340	0.073	-0.133	0.413	-64	65	0.98
**0.3**	-0.371	0.095	-0.138	0.466	-73	78	0.93
**0.4**	-0.379	0.117	-0.131	0.496	-80	88	0.91
**0.5**	-0.386	0.117	-0.134	0.503	-87	97	0.90
**0.6**	-0.398	0.139	-0.130	0.537	-93	104	0.89
**0.7**	-0.413	0.143	-0.134	0.557	-98	109	0.90
**0.8**	-0.415	0.151	-0.132	0.566	-102	115	0.89
**0.9**	-0.432	0.149	-0.142	0.581	-107	122	0.88

Table S3: Electrochemical characteristics of a redox probe $Ru(NH_3)_6Cl_3$ on RDGCE/MXene/CB_e

**MXene/CB_e**							
**Scan rate / V s^{-1}**	E_{pc} **/ V**	E_{pa} **/ V**	E^0 **/ V**	ΔE **/ V**	i_{pc} **/ µA**	i_{pa} **/ µA**	i_{pc} **/** i_{pa}
**0.1**	-0.205	-0.132	-0.169	0.073	-69	58	1.19
**0.2**	-0.208	-0.132	-0.170	0.076	-100	87	1.14
**0.3**	-0.210	-0.134	-0.172	0.076	-124	111	1.11
**0.4**	-0.210	-0.134	-0.172	0.076	-147	131	1.12
**0.5**	-0.213	-0.134	-0.173	0.078	-166	150	1.10
**0.6**	-0.210	-0.134	-0.172	0.076	-183	168	1.09
**0.7**	-0.210	-0.132	-0.171	0.078	-201	185	1.09
**0.8**	-0.213	-0.134	-0.173	0.078	-217	202	1.08
**0.9**	-0.213	-0.132	-0.172	0.081	-232	218	1.07

Table S4: Electrochemical characteristics of a redox probe $Ru(NH_3)_6Cl_3$ on RDGCE/MXene/SB

**MXene/SB**							
**Scan rate / V s^{-1}**	E_{pc} **/ V**	E_{pa} **/ V**	E^0 **/ V**	ΔE **/ V**	i_{pc} **/ µA**	i_{pa} **/ µA**	i_{pc} **/** i_{pa}
**0.1**	-0.158	-0.085	-0.121	0.073	-65	58	1.11
**0.2**	-0.160	-0.085	-0.122	0.076	-90	87	1.04
**0.3**	-0.160	-0.085	-0.122	0.076	-111	109	1.01
**0.4**	-0.160	-0.085	-0.122	0.076	-128	128	1.00
**0.5**	-0.160	-0.085	-0.122	0.076	-143	144	0.99
**0.6**	-0.163	-0.085	-0.124	0.078	-157	158	0.99
**0.7**	-0.163	-0.085	-0.124	0.078	-170	171	1.00
**0.8**	-0.165	-0.082	-0.124	0.083	-182	183	0.99
**0.9**	-0.165	-0.082	-0.124	0.083	-194	195	0.99

Table S5: Electrochemical characteristics of a redox probe $Ru(NH_3)_6Cl_3$ on RDGCE/MXene/SB_e

**MXene/SB_e**							
**Scan rate / V s^{-1}**	**E_{pc} / V**	**E_{pa} / V**	**E^0 / V**	**ΔE / V**	**i_{pc} / µA**	**i_{pa} / µA**	**i_{pc} / i_{pa}**
**0.1**	-0.158	-0.085	-0.121	0.073	-64	61	1.06
**0.2**	-0.160	-0.085	-0.122	0.076	-89	89	1.00
**0.3**	-0.160	-0.085	-0.122	0.076	-109	111	0.98
**0.4**	-0.163	-0.085	-0.124	0.078	-126	129	0.98
**0.5**	-0.163	-0.082	-0.122	0.081	-141	145	0.98
**0.6**	-0.163	-0.082	-0.122	0.081	-155	158	0.98
**0.7**	-0.163	-0.080	-0.121	0.083	-168	172	0.98
**0.8**	-0.165	-0.080	-0.122	0.085	-180	184	0.98
**0.9**	-0.168	-0.080	-0.124	0.088	-191	195	0.98

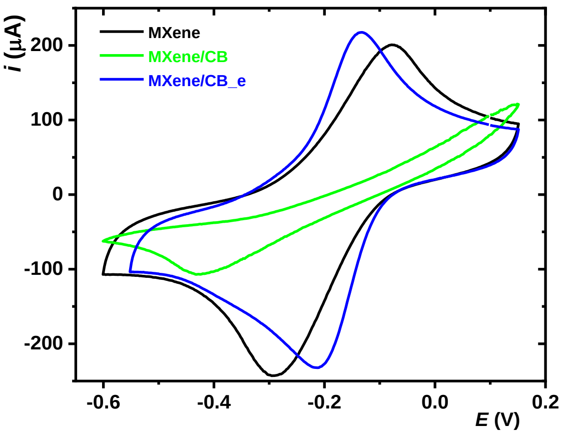

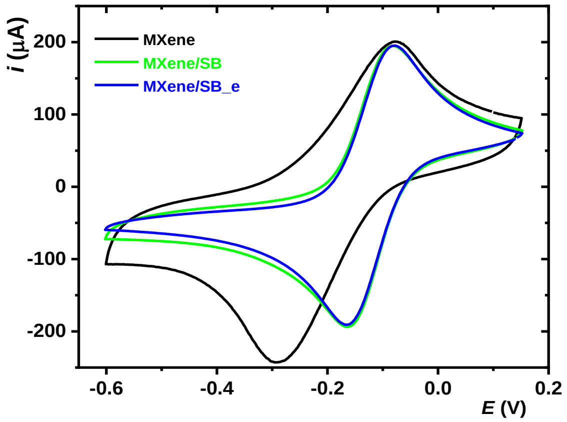

Figure S4: Electrochemical behaviour of $Ru(NH_3)_6^{3+}$ investigated on MXene and zwitterion modified MXene interfaces. CV was run at a sweep rate of 900 mV s^{-1}. For other conditions, see **Fig. S3**.

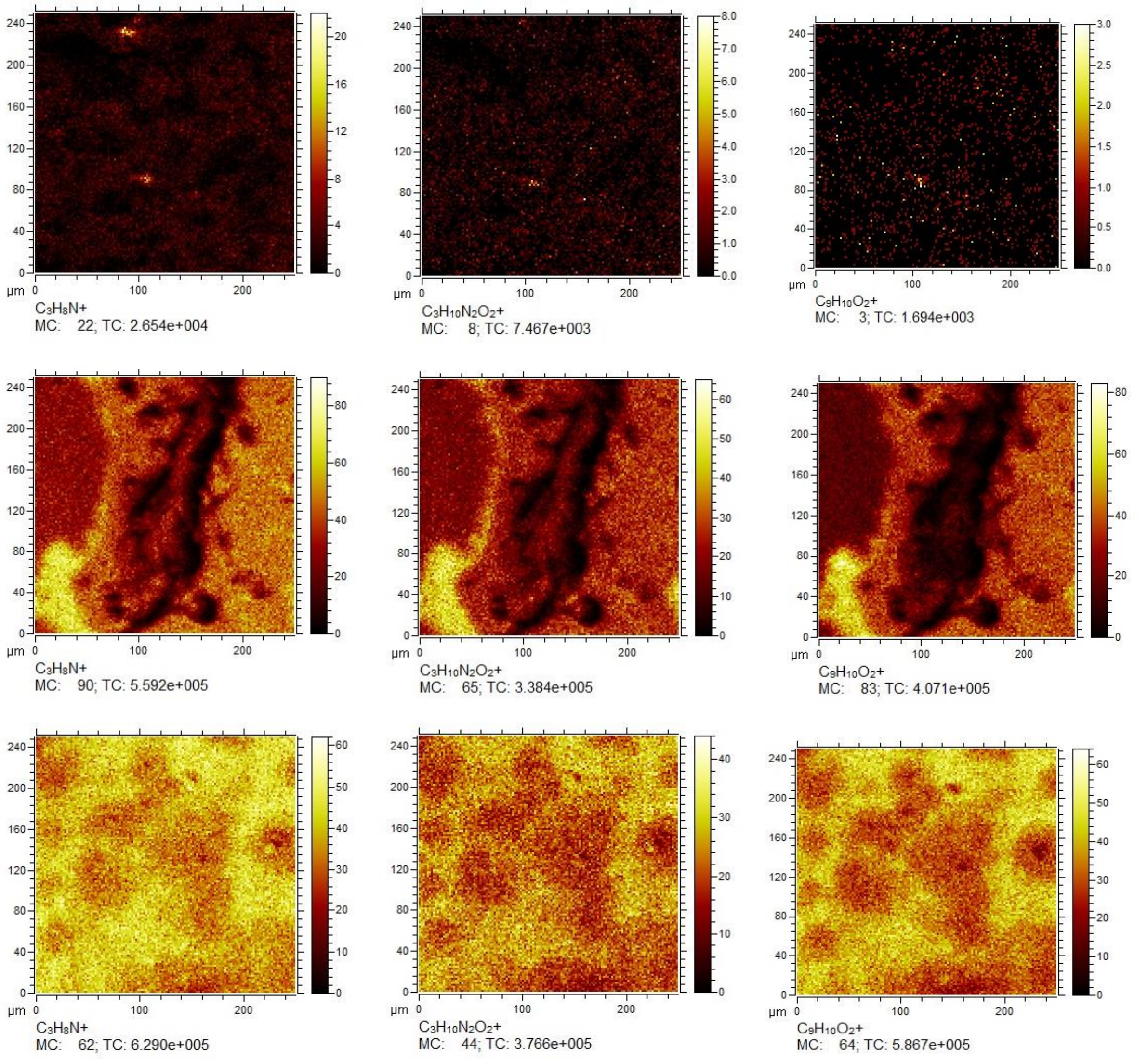

Figure S5: SIMS experiment in a positive mode showing SIMS 2D images for three selected fragments for MXene (upper row); MXene/CB (middle row) and MXene/CB_e (lower row).

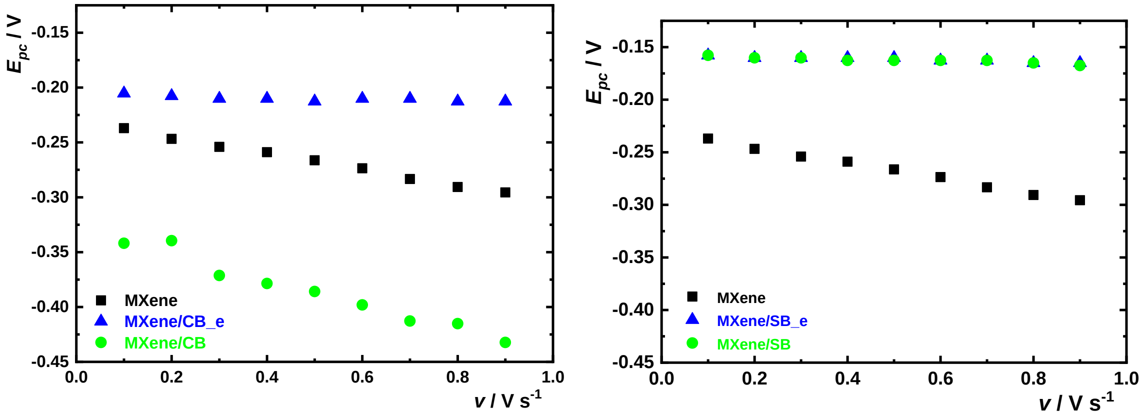

Figure S6: Electrochemical behaviour of $Ru(NH_3)_6^{3+}$ investigated on MXene and zwitterion modified MXene interfaces with a plot E_{pc} *vs.* scan rate. For other experimental conditions see **Fig. S3**.

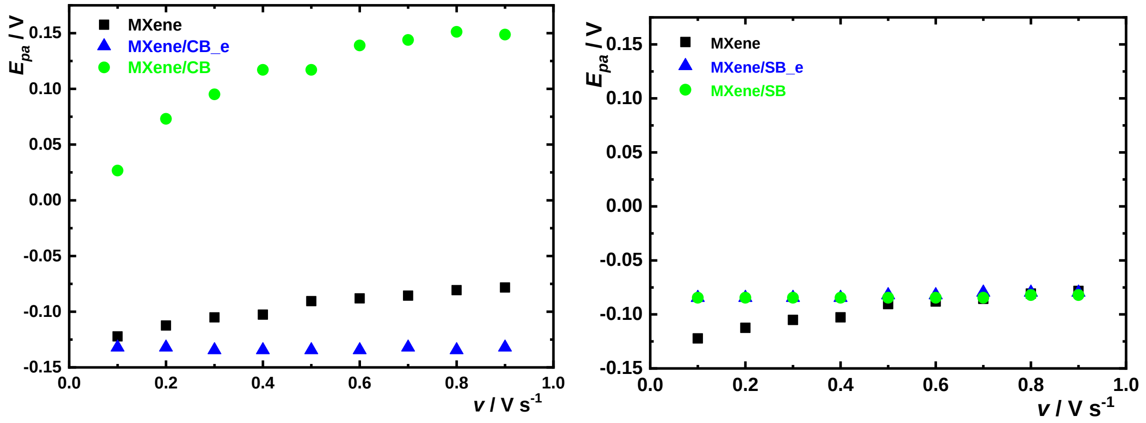

Figure S7: Electrochemical behaviour of $Ru(NH_3)_6^{3+}$ investigated on MXene and zwitterion modified MXene interfaces with a plot E_{pa} *vs.* scan rate. For other experimental conditions see **Fig. S3**.

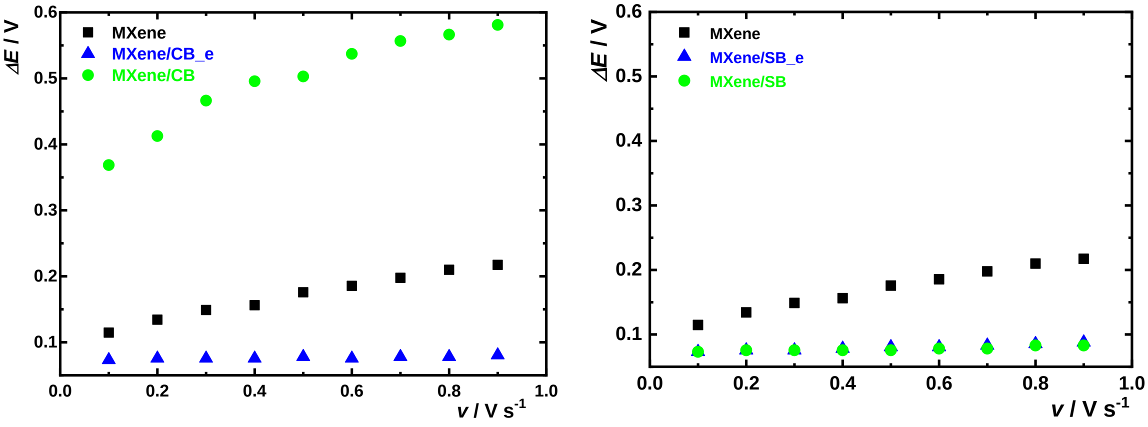

Figure S8: Electrochemical behaviour of $Ru(NH_3)_6^{3+}$ investigated on MXene and zwitterion modified MXene interfaces with a plot ΔE *vs.* scan rate. For other experimental conditions see **Fig. S3**.

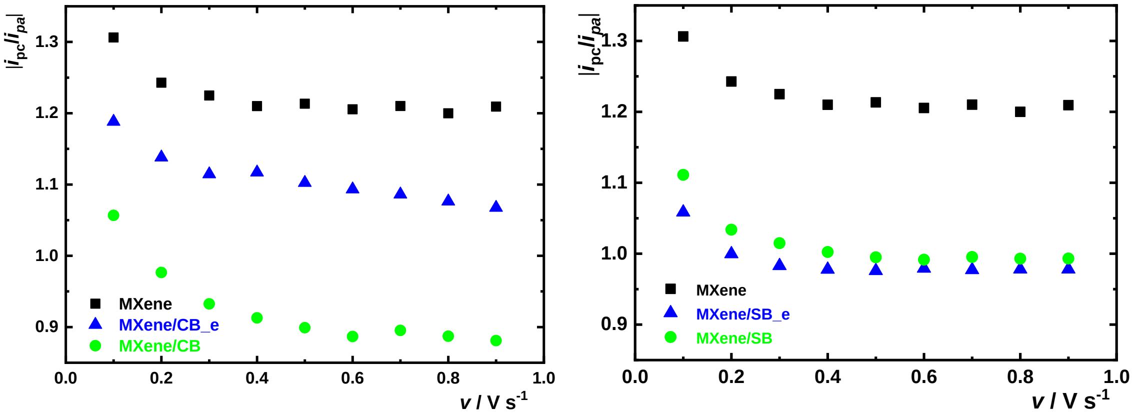

Figure S9: Electrochemical behaviour of $Ru(NH_3)_6^{3+}$ investigated on MXene and zwitterion modified MXene interfaces with a plot $|i_{pc}/i_{pa}|$ *vs.* scan rate. For other experimental conditions see **Fig. S3**.

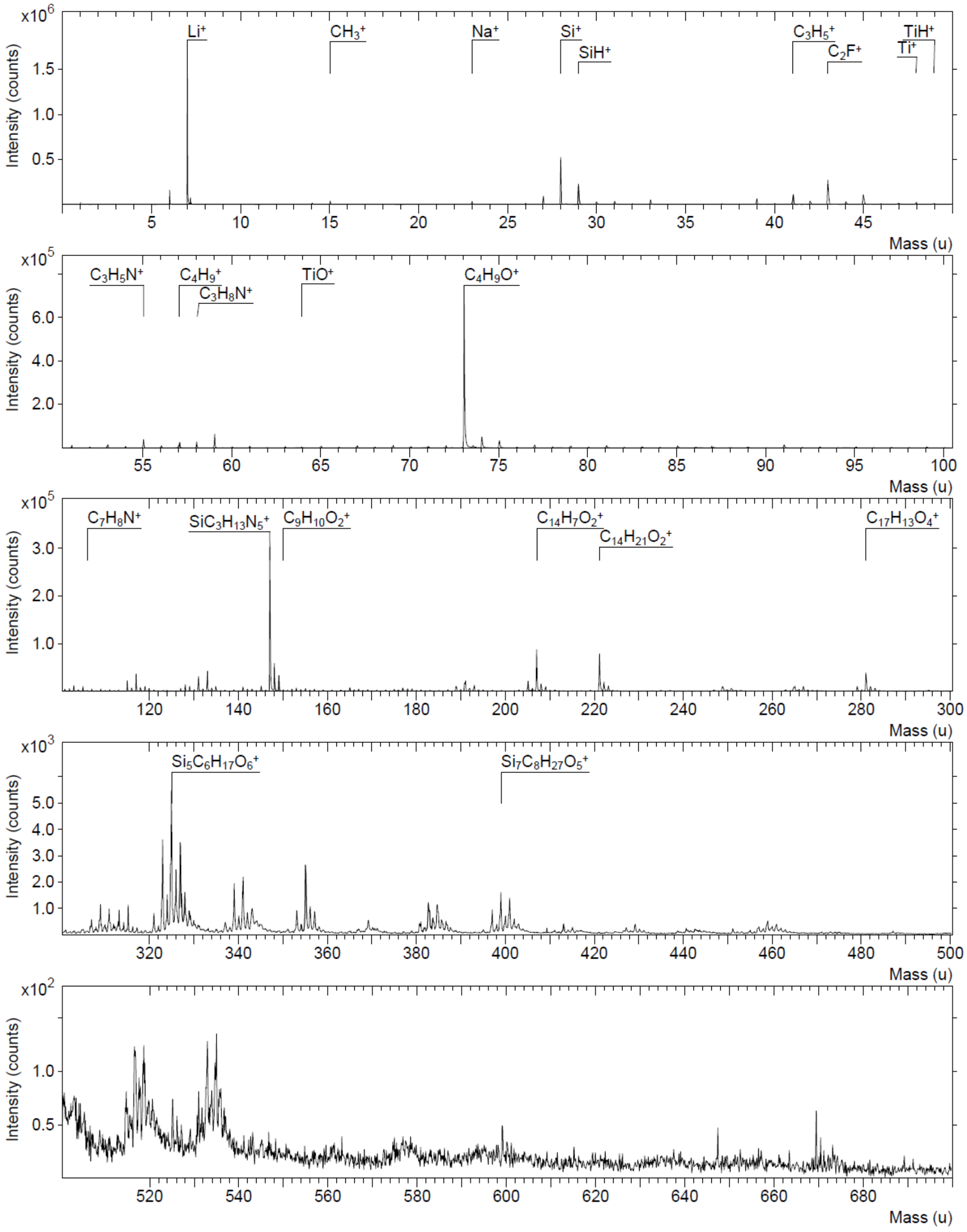

Fig. S10: SIMS spectrum of MXene in a positive polarity.

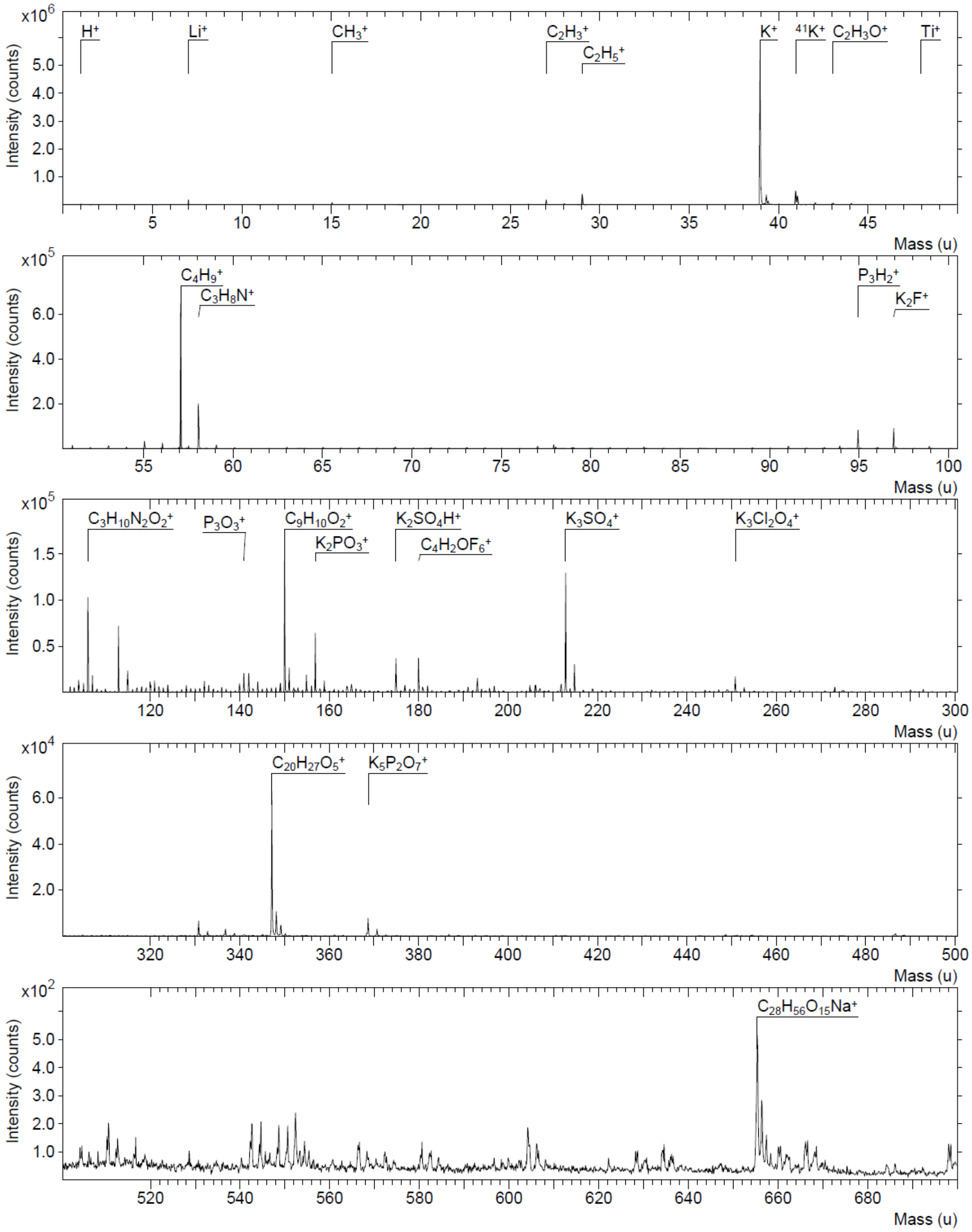

Fig. S11: SIMS spectrum of MXene/CB in a positive polarity.

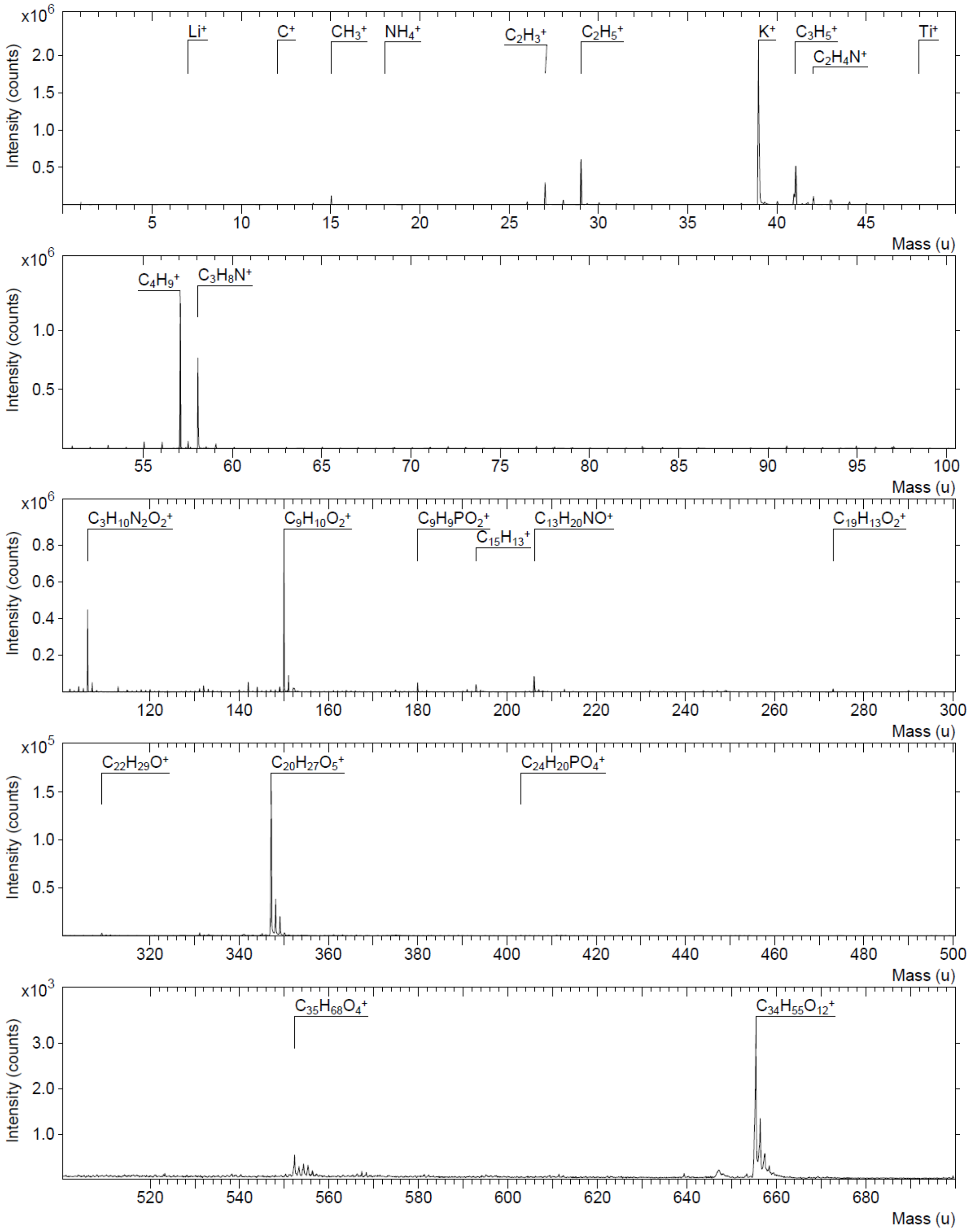

Fig. S12: SIMS spectrum of MXene/CB_e in a positive polarity.

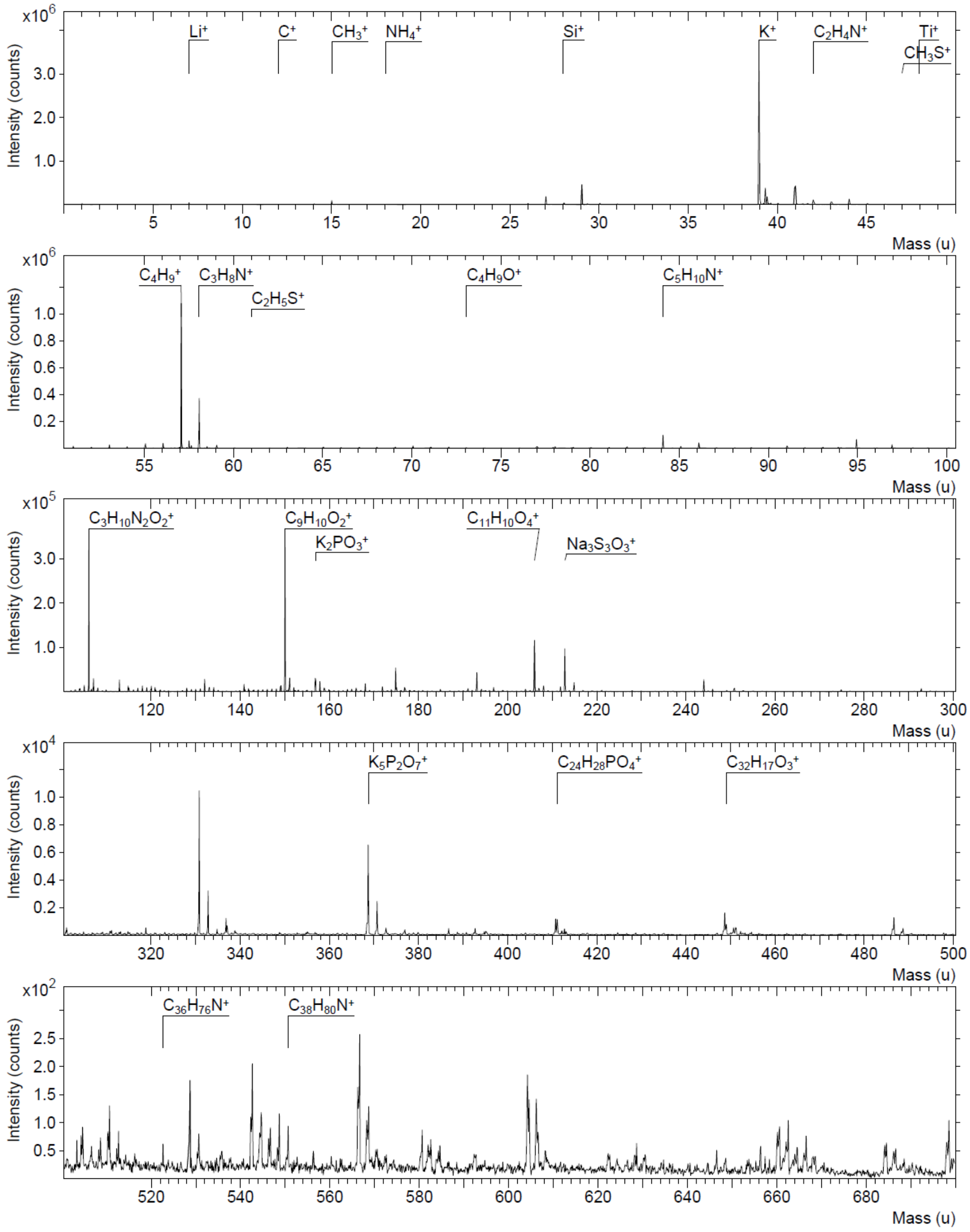

Fig. S13: SIMS spectrum of MXene/SB in a positive polarity.

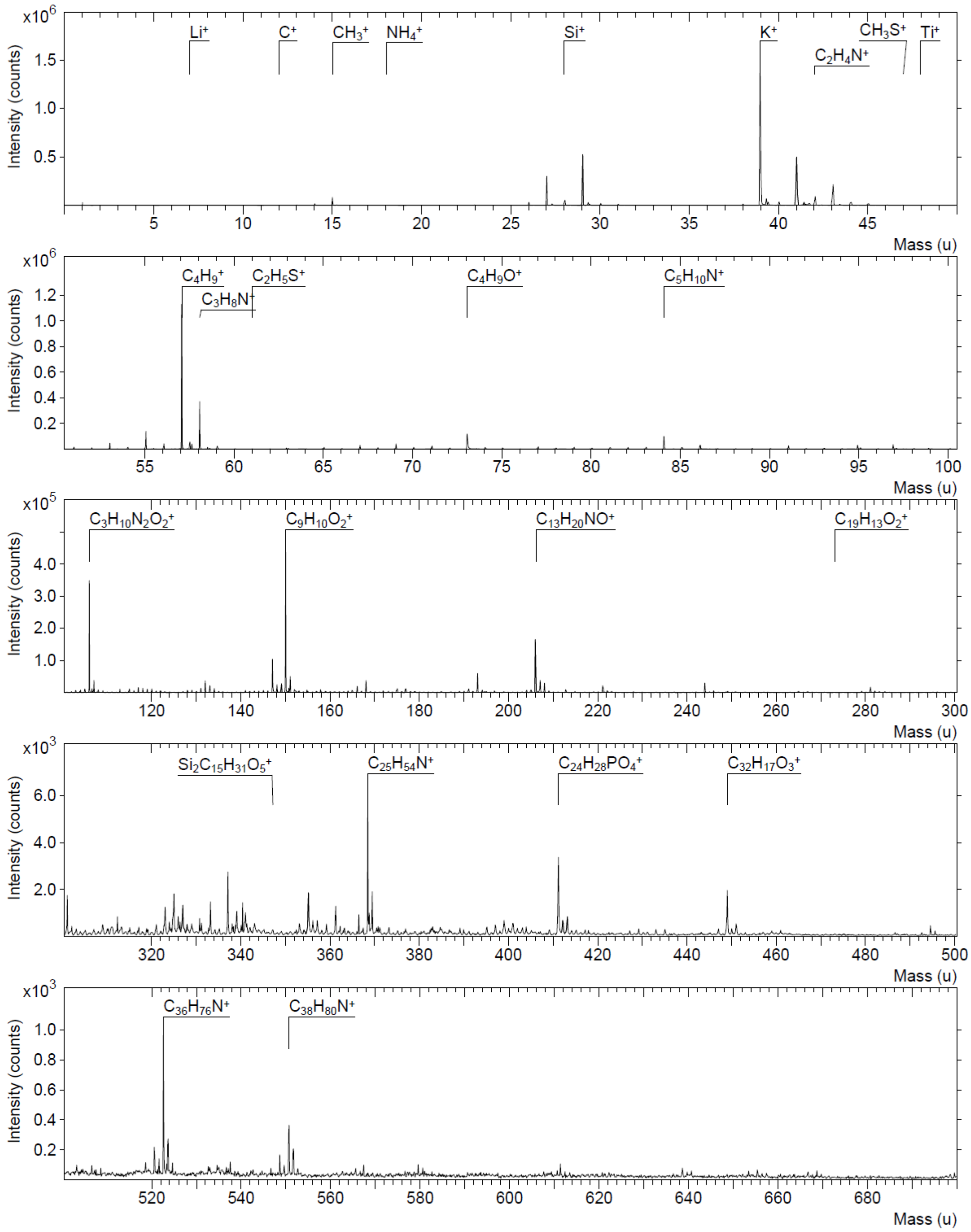

Fig. S14: SIMS spectrum of MXene/SB_e in a positive polarity.

References

1. Zhang, T.; Pan, L.; Tang, H.; Du, F.; Guo, Y.; Qiu, T.; Yang, J. Synthesis of two-dimensional Ti3C2Tx MXene using HCl+LiF etchant: Enhanced exfoliation and delamination. *J. Alloys Compd.* **2017**, *695*, 818–826.

2. Alhabeb, M.; Maleski, K.; Anasori, B.; Lelyukh, P.; Clark, L.; Sin, S.; Gogotsi, Y. Guidelines for Synthesis and Processing of Two-Dimensional Titanium Carbide ($Ti_3C_2T_x$ MXene). *Chem. Mater.* **2017**, *29*, 7633–7644.
